# Adverse Childhood Experiences, Mindfulness, and Grit in College Students in China

**DOI:** 10.3389/fpsyg.2022.891532

**Published:** 2022-05-30

**Authors:** Shannon P. Cheung, Bin Tu, Chienchung Huang

**Affiliations:** ^1^School of Social Work, Rutgers, The State University of New Jersey, New Brunswick, NJ, United States; ^2^Guangdong Research Center for NPO, Guangdong University of Foreign Studies, Guangzhou, China

**Keywords:** adverse childhood experience, college students, emerging adults, grit, mindfulness

## Abstract

This study investigated the effect of ACEs and COVID-19 on grit and whether this effect is mediated by mindfulness. Although current scholarship has found that adverse childhood experiences (ACEs) have harmful consequences to individuals across the life span, less is known about the relationship between ACEs and grit. Grit is predictive of educational success and subjective wellbeing. A cross-sectional online survey administered to junior and senior students from 12 universities spread across China was conducted from September 20, 2020 to October 5, 2020. The universities were selected from geographically diverse regions of China to ensure a diverse sample. We received 1,871 completed responses from 2,229 invited students. The survey response rate was 83.9%. The results indicated that ACEs had significantly negative effects on grit, while mindfulness had significantly positive effects on grit. Once controlling for level of mindfulness, the effects of ACEs on grit largely reduced and became insignificant. The findings of this research indicate that mindfulness has a significant mediational effect on the relation between ACEs and grit and call for mindfulness-based interventions for enhancing grit for the population at risks.

## Introduction

Globally, almost two-thirds of youth have identified experiencing at least one adverse childhood experience (ACE) (Carlson et al., 2019). Over 20% of study participants in the original ACE study conducted in the United States reported experiencing three or more ACEs (Center for Disease Control and Prevention [CDC], n.d.). ACEs are categorized into three categories of events–abuse, neglect, and household challenges–that occurred within an individual’s first 18 years of life (CDC, n.d.). ACEs include harmful acts perpetrated against children, as well as familial and socioenvironmental influence that surround children. In their groundbreaking study, Felitti et al. (1998) found that ACEs are predictive of several social and health issues in adulthood. Since then, the effects of ACEs have been studied in individuals at various phases of the human life span (Brown et al., 2010; Isohookana et al., 2013; Crandall et al., 2020; Elmore et al., 2020). Parental incarceration, for example, has been reported to create significant challenges for children and families, such as maintaining parent-child relationships, throughout childhood and adolescence (Correa et al., 2021). As well, the co-occurrence of multiple ACEs is a risk factor for a myriad of health conditions, including diabetes, chronic lung disease, depression, and suicidality, as well as risky behaviors like early sexual initiation and violence perpetration (see Hughes et al., 2017 for review).

Given the deleterious effects of ACEs, it comes as no surprise that the attention of scholars has increasingly been directed at constructs such as resilience and grit. In the present study, we focus on grit, or “perseverance and passion for long-term goals” (Duckworth et al., 2007, p. 1087). Initially studied as a predictor of academic and professional success (Duckworth et al., 2007), grit has made its way into health and wellbeing studies. For example, grit has a buffering effect for suicidal ideation risk (Kleiman et al., 2013) as well as protective effects against peer victimization and problematic video gaming (Li and Zhu, 2020). Additionally, grit is inversely associated with psychological distress (Datu et al., 2018), substance use (Guerrero et al., 2016), and depression (Musumari et al., 2018). More generally, grit is a strong, positive predictor of subjective wellbeing (Singh and Jha, 2008).

Since grit is predictive of psychological and subjective wellbeing, the field requires understanding on possible points of intervention to enhance grit within vulnerable populations. Studies have identified that parent characteristics (e.g., employment and parenting style) and individual traits (e.g., goal commitment and self-efficacy) may affect adolescents’ grit (Guerrero et al., 2016; Datu, 2017; Tang et al., 2019). Mindfulness, a mental state involving purposeful attention and non-judgmental reactions (Kabat-Zinn, 2003), is also a positive predictor of grit (Raphiphatthana et al., 2018). Mindfulness is comprised of two key components: mindful attention and mindful metacognition. The former regulates attention by emphasizing awareness of the present moment, while the latter consists of intentionally detaching oneself from monitoring value-laden thoughts and feelings that may arise (Bishop et al., 2004; Keng et al., 2011; Reina and Kudesia, 2020). Studies have shown that mindfulness can bolster academic performance (Lu et al., 2017; Caballero et al., 2019), improve social and emotional competence (Schonert-Reichl and Lawlor, 2010; Klingbeil et al., 2017), and mitigate emotional and behavioral problems (van de Weijer-Bergsma et al., 2012; Huang et al., 2019).

Recent grit studies center experiences of college students (Bono et al., 2020; Sulla et al., 2022; Yang et al., 2022) during the COVID-19 pandemic, which has presented a major threat to global public health (Ali et al., 2020; Fauci et al., 2020; Wu et al., 2020). Findings showed that stress and psychological distress were high in college students during the pandemic, but grit may serve as a protective factor to reduce the effects of stress and psychological distress on wellbeing and to improve self-efficacy and academic performance (Bono et al., 2020; Sulla et al., 2022; Yang et al., 2022).

In short, previous studies point out the importance of grit for college students during the pandemic, however, there remains a need to examine how ACEs affect grit and whether mindfulness mediates the association between ACEs and grit in college students in China during the pandemic.

### Theoretical Framework

The guiding theoretical framework for this study, trauma theory, explains that traumatic experiences may negatively impact psychological wellbeing *via* the development of three symptom clusters (hyperarousal, constriction, and intrusion) (Herman, 1992). The first, hyperarousal, is one of the hallmark symptoms of post-traumatic stress disorder (PTSD). Hyperarousal describes the self-protective activation of an individual’s sympathetic nervous system by a traumatic memory. When an individual lives in a constant or chronic state of hyperarousal, they experience a prolonged state of vigilance that is challenging to regulate. Another set of symptoms, called “constriction,” may co-occur with hyperarousal. During conscription, an individual may become unresponsive to stimuli. This includes physiological, emotional, and cognitive unresponsiveness. Functionally, this state may be protective, as individuals’ unresponsiveness can help them avoid painful trauma responses. At the same time, however, intrusion symptoms can disrupt this state, forcing an individual to relive their traumatic experiences through images and sensations associated with the original experience. Notably, this occurs in nightmares or as flashbacks. These three trauma symptom clusters have been reported to disrupt individuals’ schemas about safety and trust by challenging their senses of control, connection, and meaning in life (Herman, 1992; Briere, 2019).

Contextualizing ACEs within trauma theory (Herman, 1992), this study positions ACEs as traumatic events that can have significant and persistent consequences throughout one’s life (Herrenkohl et al., 2013; Weder et al., 2014; Bryan, 2019; Mosley-Johnson et al., 2019). Individuals with ACEs may experience constriction by dissociating from triggering circumstances to cope with overwhelming reactions based in a state of hyperarousal. Long-term dissociation may affect individuals’ mindfulness through the restriction of awareness and attention to the current moment (Bishop et al., 2004; Zerubavel and Messman-Moore, 2015; Bolduc et al., 2018). As Herman (1992) suggests, trauma can leave individuals without a strong sense of connection to others or of meaning in life, overlapping with the common depressive symptom of diminished passion in life. Along the same lines, trauma is associated with escalated risky behaviors (e.g., substance use and delinquency), which can reduce one’s sense of perseverance when achieving goals and ambitions. Both, passion and perseverance are key dimensions of grit which may be negatively associated with ACEs. Therefore, ACEs are expected to reduce individual grit through the reduction of mindfulness. We hypothesized that ACEs would be negatively associated with grit in Chinese college students and that this relation would be mediated by a negative relation between ACEs and students’ mindfulness. We further hypothesized that the categories of ACEs may be differentially associated with the two dimensions of grit. Specifically, based on past findings by Datu et al. (2016) and Datu (2017), it was expected that abuse and neglect would be negatively associated with perseverance of effort.

## Materials and Methods

### Data and Sample

Data came from an anonymous online survey administered to junior and senior students in universities in China. The inclusion criteria were that participants had to be (a) social science students and (b) in their junior or senior year of college. The sample was limited to junior and senior students to assess the experiences of students who had attended university for at least 1 year prior to the pandemic. The sampling procedure was designed to reach a large and geographically diverse sample that would be sufficient to conduct multivariate analysis. Twelve leading universities were selected across the northern, eastern, southern, western and central regions of China. Once universities were selected, we reached out to each schools’ social science department, yielding a sampling frame of 2,229 students. In September 2020, all 2,229 students were invited to participate in the study, with reminders for survey completion sent out 3 and 7 days after the initial invitation. Students were informed that participation was voluntary and could be discontinued at any time. Surveys were closed in early October 2020. We received 1,881 responses. After excluding incomplete cases, the final analytic sample contained 1,871 students. The survey response rate was approximately 83.9%. This study was approved by the research review committee at one of the co-authors’ university and included an informed consent process.

### Measures

#### Grit

Grit was measured using Duckworth and Quinn’s (2009) 8-item Short Grit Scale (Grit-S). The scale is comprised of two subscales: *perseverance of effort* and *consistency of interests*, hereafter referred to simply as *perseverance* and *consistency.* In collectivist contexts (e.g., the Philippines; China), only perseverance of effort may be salient to grit (Datu et al., 2016). Perseverance is demonstrated by an individual striving harder to accomplish their goals, despite challenges and hardships, while consistency of interests is demonstrated by steady long-term interest in a project or goal. Grit-S items ask about respondents’ intrapersonal competencies and the degree to which they can maintain interest, focus, and perseverance for extended periods. Negatively worded items were recoded so that higher scores would be indicative of greater levels of grit. Average item scores, ranging 1–5, were computed to represent grit and subscale scores. In this study, the scale had a Cronbach’s alpha of 0.72. The perseverance and consistency subscales had Cronbach’s alpha values of 0.83 and 0.69, respectively.

#### Mindfulness

We measured mindfulness using the Mindful Attention Awareness Scale (MAAS) (Brown et al., 2011). The 15-item scale asks participants to indicate frequencies at which they experience different behaviors, thoughts, and behaviors over the course of the past 4 weeks. Respondents could answer 1 (almost never) to 6 (almost always). Item scores were reverse-coded so that higher scores were representative of greater mindfulness. Responses were summed to represent the mindfulness score, ranging 14–90. The Chinese version of MAAS has been found to be valid and reliable for Chinese populations (Deng et al., 2011; Huang et al., 2019). In our study, the Cronbach’s alpha was 0.90.

#### Adverse Childhood Experiences

Adverse childhood experiences were measured by the ACE questionnaire (CDC, n.d.). 10 items measured the occurrence of ACEs in three categories–abuse (3 items), neglect (2 items), and household challenges (5 items)–prior to age 18. The sum of affirmative answers represented the ACE score; higher scores indicate more ACEs. We also calculated scores by ACE categories.

#### Socioeconomic Characteristics

We collected information on students’ age, sex, ethnicity, household registration (HR), and proximity to COVID-19. Students were also asked to report on their parents’ marital status and educational attainment, as well as number of family members they had, family income in the last year, and whether their family received welfare in the last year. We measured *proximity to* COVID-19 by asking students to indicate whether they knew of any family members or friends who had tested positive for or died as a result of COVID-19. Finally, we considered that each college’s characteristics may affect students’ grit and accounted for this by controlling for college-fixed effects.

### Analyses

Analyses began with descriptive analysis to examine the distribution of our variables of interest. This was followed by ordinary-least-square (OLS) regression analysis, which estimated the net effects of our independent variables on grit, controlling for socioeconomic characteristics. The following equation represents the analytic model:


$$Y_{\text{i}}=\alpha_{\text{i}}+\beta_{1}*\chi_{\text{i}}+{\text{C}}_{\text{i}}+\varepsilon_{\text{i}},$$

where *Y* i is subject i’s grit; α_i_ is the individual constant; χ is a vector of subject i’s mindfulness, ACEs, and socioeconomic characteristics; C_i_ is the college-fixed effects of subject i’s college, taken to be constant across individual colleges; β is a vector of regression coefficients; and ε_i_ is the cross-section error component. By incorporating a term for college-fixed effects, this analytic model controls for differences across the 12 colleges from which we sampled. Analyses were conducted using STATA software 16.0.

## Results

### Descriptive Statistics

Results of descriptive statistics for the variables of interest and socioeconomic characteristics are presented in Tables 1, 2, respectively. The sample had an average grit score of 3.07 (*SD* = 0.44). Perseverance and consistency scores averaged 3.28 and 2.86, respectively. On average, the students in our sample had a mindfulness score of 59.61 (*SD* = 10.84). Scores ranged from 15 to 90. 35.16% of the sample reported having experienced at least one ACE, while 8% reported that they had experienced at least three. The sample had an average ACE score of 0.69 (*SD* = 1.28). Subscale score averages are also presented in Table 1.

**TABLE 1:** Level of grit, mindfulness, adverse childhood experience, and COVID-19 infection.

	Mean (S.D.)
Grit (1–5)	3.07 (0.44)
Perseverance (1–5)	3.28 (0.72)
Consistency of effort (1–5)	2.86 (0.63)
Mindfulness (15–90)	59.61 (10.84)
Adverse childhood experience (%)	
Occurrence (No = 0, Yes = 1)	35.16
Three types or more	8.44
Adverse childhood experience (0–10)	0.69 (1.28)
Abuse (0–3)	0.28 (0.63)
Neglect (0–2)	0.15 (0.41)
Household challenge (0–5)	0.26 (0.61)

*N = 1871.*

**TABLE 2:** Descriptive statistics of socioeconomic variables.

	Mean (S.D.)
Gender (%)	
Female	66.97
Male	33.03
Age	20.62 (0.96)
Household registration (%)	
Rural	38.70
City, rural before	8.93
City	52.37
Grade (%)	
Junior	60.72
Senior	39.28
Ethnicity (%)	
Han	89.36
Others	10.64
Parent marital status (%)	
Married	89.04
Separated	0.80
Divorced	6.89
Widowed	2.35
Others	0.91
Parent highest education achievement (%)	
Elementary school and below	6.90
Junior high school	28.11
High school	25.17
College and above	39.82
Family income	90990 (122030)
Welfare status	
No	74.72
Yes	25.28
Number of family members	3.87 (1.16)
COVID-19 infection in family and friends (%)	
No	99.14
Infected	0.48
Dead	0.37
College (%)	
College 1	7.11
College 2	9.57
College 3	6.25
College 4	10.85
College 5	10.15
College 6	7.06
College 7	6.41
College 8	11.54
College 9	11.12
College 10	2.46
College 11	6.89
College 12	10.58

*N = 1871.*

Female students comprised two-thirds of the sample, which is reflective of the social science student population across Chinese academic institutions. The sample’s average age was 20.62. Nearly 90% of the sample was of Han ethnicity. About half (52.37%) held city HR, followed by rural HR (38.60%), and city with prior rural HR (8.93%). Most students (89.04%) reported that their parents were married. 6.89% reported that their parents were divorced. About 40% reported that their parents’ highest educational attainment was college and above, followed by junior high school (28.11%), high school (25.17%), and elementary school (6.9%). Family income averaged at 90,990 RMB, or about 13,580 USD, in the past year (*SD* = 122,030 RMB or 18,170 USD). About one-quarter of the sample reported that their families were recipients of social welfare (e.g., low-income assistance and food subsidies) in the past year. Students had an average of 3.87 family members. Due to low occurrence of students reporting having family or friends who had been infected with COVID-19 (0.5%) or who had died of COVID-19 (0.4%), we aggregated both to create a single category for analysis. Students from each college ranged from ranged from 2.5 to 11.5% of the analytic sample, reflecting the size of the student body in their respective schools.

### Multivariate Analyses

Table 3 presents standardized estimates of grit, estimated by OLS regression and predicted by two different models. Model 1 includes ACE scores and student socioeconomic characteristics. We added mindfulness in Model 2 to test its mediational effect. The results of Model 1 show that ACEs are a significant and negative predictor of grit (*B* = −0.09). Grit was also positively associated with age. The adjusted R-square of Model 1 was 0.03 and increased to 0.18 in Model 2. In Model 2, mindfulness was a significant predictor of grit (*B* = 0.41), while the ACE estimate lost statistical significance. Other estimates produced by Model 2 are similar to those of Model 1. The increase in adjusted *R*-square values between the models, coupled with the large estimate of mindfulness, suggests that much of the variance in grit within the sample was associated with students’ mindfulness and that mindfulness fully mediated the effects of ACEs on grit.

**TABLE 3:** Regression analysis of grit.

	Model 1			Model 2		
	B	S. E.	*P*	B	S. E.	*P*
Adverse childhood experience (Score)	−0.09	0.01	***	0.00	0.01	
Mindfulness	—	—		0.41	0.01	***
Female	0.04	0.02	+	0.04	0.02	+
Age	0.10	0.01	**	0.07	0.01	**
Household registration: city, rural before	−0.03	0.04		−0.03	0.04	
Household registration: city	0.03	0.03		0.01	0.03	
Junior	0.01	0.03		0.01	0.02	
Han	0.00	0.03		−0.01	0.03	
Married	−0.02	0.03		0.01	0.03	
Junior high school	−0.01	0.04		−0.02	0.04	
High school	0.01	0.05		−0.01	0.04	
College and above	0.02	0.05		−0.02	0.05	
Family income	−0.03	0.01		−0.02	0.01	
Welfare status	0.03	0.03		0.03	0.02	
Number of family members	−0.02	0.01		−0.01	0.01	
COVID-19 infection in family and friends	−0.01	0.11		0.03	0.10	
College fixed effects	Yes			Yes		
Adjusted *R*-square	0.03			0.18		

N = 1871. +p < 0.10, **p < 0.01, ***p < 0.001.

We conducted several tests of robustness, the results of which are displayed in Table 4. These tests differ from those of Table 3 in that we utilized different specifications of the ACE variable each time: total ACE score (Specification 1, same as the one in Table 3); any ACE occurrence (Specification 2); and occurrence of three more ACEs (Specification 3). Specifications 4, 5, and 6 regressed the ACE categories–abuse, neglect, and household challenges, respectively–onto grit. Each iteration of the analysis included the controls used in the previous models. For simplicity, Table 4 presents only standardized estimates of ACE and mindfulness, as all other estimates were similar to those reported in Table 3. In Specifications 1–5, ACEs negatively affected grit, but when mindfulness was added, these estimates were no longer statistically significant. Any occurrence (Specification 2) and the occurrence of three or more ACEs (Specification 3) had negative effects on grit in Model 1 (*B* = −0.09 and −0.07, respectively). These estimates lost statistical significance in Model 2. Here, mindfulness was a significant and positive predictor of grit (*B* = 0.41). Similarly, analyses using Specification 4 and Specification 5 found that abuse and neglect negatively affected grit (*B* = −0.09 and −0.06, respectively), but in Model 2, mindfulness fully mediated these effects. Specification 6, household challenges, had no significant effects on grit in Model 1.

**TABLE 4:** Robust tests of adverse childhood experiences (ACE) on grit.

	Model 1			Model 2		
	B	S. E.	*P*	B	S. E.	*P*
ACE Scale						
Specification 1						
ACE (Score)]	−0.09	0.01	***	0.00	0.01	
Mindfulness	–	–		0.41	0.01	***
Specification 2						
Any occurrence	−0.09	0.02	***	−0.02	0.02	
Mindfulness	–	–		0.41	0.01	***
Specification 3						
Three or more occurrences	−0.07	0.04	**	−0.02	0.03	
Mindfulness	–	–		0.41	0.01	***
Three dimensions						
Specification 4						
Abuse	−0.09	0.02	***	−0.02	0.02	
Mindfulness	–	–		0.41	0.01	***
Specification 5						
Neglect	−0.06	0.02	**	0.00	0.02	
Mindfulness	–	–		0.41	0.01	***
Specification 6						
Household challenge	−0.04	0.02		0.02	0.02	
Mindfulness	–	–		0.41	0.01	***

N = 1871. **p < 0.01, ***p < 0.001.

Tables 5, 6 present robustness tests regressing each grit subscale (perseverance and consistency, respectively) on ACEs. Compared to the estimates using overall grit, ACEs had larger effects on perseverance (*B* = −0.16 for Specification 1). As in previous models, mindfulness had large effects on perseverance (*B* = 0.50 for Specification 1), but, unlike previous models, it did not fully mediate the effects of ACEs. The ACE estimate reduced by 63% between the models but remained significant for Specification 1 (*p* < 0.01). Estimates for Specifications 2 and 3 reduced similarly and were both marginally significant (*p* < 0.10) in Model 2. Among the ACE categories, mindfulness only fully mediated neglect’s effect on perseverance. In Table 6, ACE score and household challenges had significant positive effects on consistency (*B* = 0.07 and 0.10, respectively), but all other specifications and mindfulness did not have any significant effects on consistency.

**TABLE 5:** Robust tests of grit subscale–perseverance.

	Model 1			Model 2		
	B	S. E.	*P*	B	S. E.	*P*
ACE Scale						
Specification 1						
Adverse childhood experience (Score)	−0.16	0.01	***	−0.06	0.01	**
Mindfulness	–	–		0.50	0.01	***
Specification 2						
Any occurrence	−0.12	0.04	***	−0.04	0.03	+
Mindfulness	–	–		0.50	0.01	***
Specification 3						
Three or more occurrence	−0.10	0.06	***	−0.04	0.05	+
Mindfulness	–	–		0.50	0.01	***
Three dimensions						
Specification 4						
Abuse	−0.13	0.03	***	−0.04	0.02	+
Mindfulness	–	–		0.50	0.01	***
Specification 5						
Neglect	−0.10	0.04	***	−0.02	0.04	
Mindfulness	–	–		0.50	0.01	***
Specification 6						
Household challenge	−0.14	0.03	***	−0.07	0.03	**
Mindfulness	–	–		0.50	0.01	***

N = 1871. +p < 0.10, **p < 0.01, ***p < 0.001.

**TABLE 6:** Robust tests of adverse childhood experiences (ACE) on grit subscale–consistency of interests.

	Model 1			Model 2		
	B	S. E.	*P*	B	S. E.	*P*
ACE Scale						
Specification 1						
Adverse childhood experience (Score)	0.07	0.01	**	0.07	0.01	**
Mindfulness	–	–		0.01	0.00	
Specification 2						
Any occurrence	0.02	0.03		0.02	0.03	
Mindfulness	–	–		0.00	0.00	
Specification 3						
Three or more occurrence	0.01	0.05		0.01	0.05	
Mindfulness	–	–		0.00	0.00	
Three dimensions						
Specification 4						
Abuse	0.02	0.02		0.02	0.02	
Mindfulness	–	–		0.00	0.00	
Specification 5						
Neglect	0.03	0.04		0.03	0.04	
Mindfulness	–	–		0.00	0.00	
Specification 6						
Household challenge	0.10	0.03	***	0.10	0.03	***
Mindfulness	–	–		0.00	0.00	

N = 1871. *p < 0.05, **p < 0.01, ***p < 0.001.

## Discussion

This study sought to investigate how ACEs affect grit in a sample of college students during the COVID-19 pandemic. We hypothesized that ACEs would be negatively associated with grit and that mindfulness would mediate this relationship. Our ACEs hypothesis was supported by the results, though the findings revealed that the extent of the effects on grit differed by ACE specification, as indicated by robustness tests. Analyses by ACE category indicated that abuse and neglect had strong effects on grit overall, while household challenges did not. This study indicates that ACEs have a small to moderate effect on grit. Regression analyses including mindfulness revealed that all specifications no longer predicted grit. This is consistent with past findings that have found that mindfulness predicts grit (Raphiphatthana et al., 2018).

The significant effects of abuse and neglect on grit may be explained by decreased sense of relatedness, defined as the extent to which a person feels accepted by social partners, including parents (Datu, 2017). Trauma theory (Herman, 1992) posits that trauma can lead to negative self-appraisal and feelings of rejection. These can in turn reduce an individual’s sense of relatedness and, therefore, grit. Previously, Datu et al. (2016) suggested that perseverance of effort may be more relevant than consistency of interests in the collectivist contexts of Asian cultures. Indeed, all ACE specifications had significant negative effects on perseverance, and when mindfulness was added to the model, only one specification was no longer significant, while two others were marginally significant. These findings may be explained by the importance of the “context-sensitive self” (Suh, 2007) and relational harmony (Markus and Kitayama, 1991) in collectivistic cultures. Consistency of interests may be irrelevant since individuals are more likely to prioritize fulfilling others’ expectations (e.g., those of their parents’) over pursuing personal interests. In doing so, individuals may see themselves as maintaining peace within the larger social unit (e.g., a family) in which they are embedded. By contrast, pursuing one’s personal interests, which may not align with the family’s best interests, may be disruptive to such relationships and can cause conflict. ACEs may negatively affect perseverance of effort *via* mental illnesses like depression (Schilling et al., 2007; Merrick et al., 2017), which can manifest in symptoms like avolition and anhedonia (Price and van Stolk-Cooke, 2015). Moreover, traumatic events can lead to low self-esteem (Stern et al., 1995), and perceived lack of competence may discourage individuals from pursuing goals when faced with adversity.

Household challenges had no significant effect on overall grit but positively estimated consistency of interests (*B* = 0.10), even when mindfulness was added. This may be a result of disrupted relationships caused by dysfunctional home environments. An individual may choose to pursue personal interests rather than tend to expectations of others due to perceived lack of acceptance and lower sense of relatedness (Datu, 2017).

Our findings, contextualized within the central values of filial piety and family in East Asian culture, suggest a number of research and practice implications for researchers and social service professionals who study and work with children and families, especially those with similar cultural traditions. Research on ACEs may benefit from understanding not just the psychological traits that ACEs can affect but also the moderators and mediators of these relations, particularly those that involve the family. For example, family functioning has been found to moderate the relation between ACEs and adolescent health and emotional wellbeing (Balistreri and Alvira-Hammond, 2016; Scully et al., 2020). Other research has found family functioning to be a mediator of mental health problems in children and adolescents. In our study, we found direct relations between ACEs and grit, although these relations varied by grit subscales. This finding may guide future family research to examine how family relationships, as well as individuals’ perceptions and appraisals of these relationships, are affected by ACEs and whether this relation mediates the relation between ACEs and grit. Finally, these results extend the work of grit scholars who have studied the construct outside of the Western context in which it was originally derived. By showing that only the perseverance of effort dimension was salient for this sample, we highlight that the originally theorized dimensions of grit may lack cross-cultural conceptual equivalence in Chinese college students (Hui and Triandis, 1985). That is, grit, with its current paradigmatic definitions and dimensions, may not necessarily have the same meaning across college students of cultural groups. Grit was originally theorized in the individualistic United States American context (Duckworth et al., 2007). Future grit research may thus explicitly examine the family-centered social and cultural norms in China and how individuals’ personal sense of relatedness to those around them and how the concept of saving face may lead them to prioritize perseverance of effort over personal interests.

Meanwhile, for social service providers who work with families in which children or adolescents have experienced ACEs, this study points to the utility of mindfulness interventions to mitigate the effects of ACEs on grit. While mindfulness in practice with individuals has proliferated significantly in past decades (Creswell, 2017), the integration of mindfulness in family therapy has been less common (Brody et al., 2018; Beaudoin and Maclennan, 2020). Brody et al. (2018) argued for the integration of mindfulness in family therapy with adolescents because of mindfulness’s ability to promote emotion regulation. We add onto this by reflecting that mindfulness also mediates the relation between ACEs and grit and emphasizing the need for mindfulness interventions in practice with children and adolescents who have experienced emotional abuse and/or neglect.

## Limitations

This study has several limitations. First, cross-sectional data can only approximate associative relations; a longitudinal design can better examine the causal relations of ACEs, grit, and mindfulness. Second, unobserved variables (e.g., academic stressors and peer support) could affect students’ grit but were not included in the study, thus affecting our estimates. Third, the sample consisted of only social science students, many of whom are female (as indicated by our sample, which was two-thirds female). The extent to which our findings can be generalized to other students, such as those from male-dominated fields, like science and technology, is unknown and requires further research. Fourth, our study may not have found effects of COVID-19 proximity on grit due to measurement error, as data were dependent on students’ recall. The COVID-19 infection rate had stabilized in China since April 2020 (Zhong and Wang, 2020), 5 months prior to the time of data collection. Research in countries where COVID-19 continues to affect daily life is warranted. Last, due to self-reporting, data were subject to reporting errors. In collectivistic cultures, face-saving may be prioritized when discussing family matters (Eriksson et al., 2017) which may be perceived as shameful. Future studies may consider applying triangulation during data collection. Despite these limitations, the present study expands upon existing grit literature.

## Conclusion

Our study examines how ACEs may predict grit during emerging adulthood and a pathway by which this occurs. Results indicated that ACEs negatively affect grit–particularly the perseverance of effort dimension–through mindfulness. This study’s strengths include its expansive tests of robustness, which provided further insight into how ACEs affect each grit dimension differently and how the effect of ACEs on grit may differ according to various specifications. Since grit is a predictor of academic and professional success and subjective and psychological wellbeing, individuals who have experienced ACEs may struggle in several domains of life from adolescence into adulthood. Our results suggest the necessity of mindfulness-based interventions to buffer the negative effects of ACEs on grit, especially for those who have experienced abuse and neglect. Such interventions can improve self-esteem (Thompson and Waltz, 2008; Randal et al., 2015), as well as resilience (Huang et al., 2019, 2020; Cheung et al., 2020), which has been found to be positively related to grit (Meyer et al., 2020). Although mindfulness interventions have yet to be examined for their effects on grit specifically, past literature has found that mindfulness and grit are positively associated (Raphiphatthana et al., 2018), and our study indicates that it is through mindfulness that ACEs affect grit. Our findings thus set up future research to continue building on our knowledge of grit, including its antecedents within family processes and dynamics, along with possible ways to bolster it in vulnerable populations.

## Data Availability Statement

The raw data supporting the conclusions of this article will be made available by the authors, without undue reservation.

## Ethics Statement

The studies involving human participants were reviewed and approved by Research Review Committee, School of Public Administration, Guangdong University of Foreign Studies. Written informed consent for participation was not required for this study in accordance with the national legislation and the institutional requirements.

## Author Contributions

SC, BT, and CH: conceptualization, methodology and software, validation, formal analysis, and writing—original draft preparation. BT and CH: resources, investigation, and data curation. All authors contributed to the article and approved the submitted version.

## Conflict of Interest

The authors declare that the research was conducted in the absence of any commercial or financial relationships that could be construed as a potential conflict of interest.

## Publisher’s Note

All claims expressed in this article are solely those of the authors and do not necessarily represent those of their affiliated organizations, or those of the publisher, the editors and the reviewers. Any product that may be evaluated in this article, or claim that may be made by its manufacturer, is not guaranteed or endorsed by the publisher.
